# Quantifying damage contributions from convective and stratiform weather types: How well do precipitation and discharge data indicate the risk?

**DOI:** 10.1111/jfr3.12491

**Published:** 2018-10-02

**Authors:** Katharina Schroeer, Mari R. Tye

**Affiliations:** ^1^ Wegener Center for Climate and Global Change (WEGC) University of Graz Graz Austria; ^2^ FWF‐DK Climate Change University of Graz Graz Austria; ^3^ Capacity Center for Climate & Weather Extremes, Mesoscale and Microscale Meteorology Laboratory National Center for Atmospheric Research Boulder Colorado; ^4^ Now at Federal Office of Meteorology and Climatology MeteoSwiss Zurich Switzerland

**Keywords:** convective precipitation, extreme events, natural hazard, nuisance events, vulnerability, weather types

## Abstract

Convective precipitation is intensifying in many regions, but potential implications of shifts in precipitation types on impacts have not been quantified. Furthermore, risk assessments often focus on rare extremes, but also more frequent hydro‐meteorological events burden private and public budgets. Here synoptic, hydrological, meteorological, and socio‐economic data are merged to analyse 25 years of damage claims in 480 Austrian municipalities. Exceedance probabilities of discharge and precipitation associated with damage reports are calculated and compared for convective and stratiform weather patterns. During April to November, 60% of claims are reported under convective conditions. Irrespective of the weather type, most of the accumulated cost links to minor hazard levels, not only indicating that frequent events are a highly relevant expense factor, but also pointing to deficiencies in observational data. High uncertainty in damage costs attributable to extreme events demonstrates the questionable reliability of calculating low‐frequency event return levels. Significant differences exist among weather types. Stratiform weather types are up to 10 times more often associated with damaging extreme discharge or precipitation, while convective weather shows the highest nuisance level contributions. The results show that changes in convective precipitation are pertinent to risk management as convective weather types have contributed significantly to damage in the past.

## 1 INTRODUCTION

Damage from extreme precipitation disrupts daily life and imposes financial burdens on private and public budgets. In Austria, damage repairs from hydro‐meteorological hazards are largely supported by a tax‐financed disaster fund. While fluvial flooding, often stemming from large‐scale precipitation, is considered in risk assessments on the national level (BMNT, 2011), also flash floods, debris flows, and landslides frequently cause damage. These events are often caused by intense localised convective precipitation on sub‐daily time scales (Aceto, Caloiero, Pasqua, & Petrucci, 2016; Llasat, Marcos, Turco, Gilabert, & Llasat‐Botija, 2016).

Contributions from convective precipitation to total damage volume are not well quantified, but knowledge of such damage potential is important because extreme convective precipitation is expected to intensify with global warming in many regions (Bao, Sherwood, Alexander, & Evans, 2017; Dai, Rasmussen, Liu, Ikeda, & Prein, 2017; Donat, Lowry, Alexander, O'Gorman, & Maher, 2016; Prein et al., 2017; Westra, Alexander, & Zwiers, 2012). The frequency of convective storms has increased over the mid‐latitudes (Feng et al., 2016; Ye, Fetzer, Wong, & Lambrigtsen, 2017), and atmospheric conditions favouring severe storms are projected to increase over central‐southern Europe (Púčik et al., 2017). Convective storms challenge risk assessments, because associated extreme precipitation and runoff are not well represented in gauge observations (Eggert, Berg, Haerter, Jacob, & Moseley, 2015; Lebel, Bastin, Obled, & Creutin, 1987; Schrooer, Kirchengast & O, 2018). There is still considerable underestimation of extreme intensities in radar estimates (Kann et al., 2015; Peleg et al., 2018). Insurance data can complement sparse observations, but come with their own set of uncertainties (Grahn & Nyberg, 2017; Punge & Kunz, 2016; Wirtz, Kron, Löw, & Steuer, 2014).

Data limitations often force regional risk assessments to focus on large‐scale precipitation patterns or to single out specific catchments or events (e.g., Boudou, Lang, Vinet, & Cœur, 2016). Bernet, Prasuhn, and Weingartner (2017) showed that surface water flooding away from watercourses, which is rarely considered in studies, contributes considerably to overall flood losses in Switzerland. The shares were particularly high in conjunction with local extreme rainfall intensities, while after long‐duration precipitation events more fluvial flood damages occurred. This suggests that damage patterns from convective precipitation are different than from stratiform events, but little is known about these differences.

Weather types, which describe the larger‐scale synoptic situation over a region, can increase confidence in the type of precipitation despite fragmentary ground observations (e.g., Prein, Holland, Rasmussen, Clark, & Tye, 2016). Weather types have been used, for example, to determine synoptic conditions favourable to landslide occurrence (Wood, Harrison, Turkington, & Reinhardt, 2016), to develop a weather based risk index for flooding and landslides in Italian regions (Messeri et al., 2015), or to identify conditions driving very rare flood events in a long historical record (De Niel, Demarée, & Willems, 2017).

Localised extreme precipitation, typical for convective weather patterns, can be perceived as rare and catastrophic from a local perspective, but the probability of occurrence increases over larger domains (e.g., Sass et al., 2012; Syed, Goodrich, Myers, & Sorooshian, 2003). Moftakhari, AghaKouchak, Sanders, and Matthew (2017) showed how exposure to less severe frequent coastal flooding, so‐called “nuisance” events (annual exceedance probability AEP > 0.5), accumulates to financial risk similar to the risk from more extreme events (AEP < 0.05). So far, attention to inland nuisance flooding has been limited and focused on stream flow rather than precipitation (Slater & Villarini, 2016). Yet, for risk management actors such as the Austrian government, financing disaster recovery through the public disaster fund, such cumulative damage contributions are highly relevant. Because risk measures in the form of exceedance probabilities are typically derived from daily observations, in which convective extremes are not well represented, convective precipitation is more likely to appear as nuisance events and its impacts may be misrepresented in regional risk analyses.

To understand how convective precipitation events have contributed to regional damage accumulation in the past and how this risk is reflected in annual exceedance probabilities of daily precipitation totals and river discharge levels, we analyse a large database of damage claims using weather types to distinguish between damage from convective and stratiform precipitation. The number and cost of reported damage claims, including damage caused by flooding, debris flows, and landslides, are assessed and associated hazard levels of discharge and precipitation are compared for distinctive patterns of convective and stratiform precipitation over the convective season from April to November.

## 2 DATA AND METHODS

### 2.1 Damage claim and municipality data

We analyse a database of 116,900 claims reported to the Austrian disaster fund (Katastrophenfonds) in 480 municipalities in southeastern Austria over 25 years (1990–2014). The data were obtained from the office of the provincial government of Styria, Austria.

The disaster fund is an ex‐ante form of risk financing financed by shares of annual income and corporate tax revenues (BMF, 2016). While ~70% of this fund supports preventive measures such as torrent and avalanche control, emergency services and compensation for uninsured recovery activities after exceptional events are also covered (OECD, 2014). On average, 20–30% of damage is reimbursed, with up to 50% for building damages. Payouts are only to be used to restore functionality. The types of damage eligible for reimbursement include direct damages to buildings and inventory, private roads, meadows, harvest and livestock, forest soil and roads, but exclude cars, luxury or hobby items as well as any consequential damages such as business operation losses. Before an individual can claim excess damage from the disaster fund, insured losses must be deducted from the total loss. Unfortunately, these data are not available (c.f. Prettenthaler & Vetters, 2009). There is no compulsory hazard insurance in Austria, and insurers respond to adverse selection through restricting coverage and charging expensive premiums (Holub & Fuchs, 2009). The combination of tax‐based governmental relief and adverse selection leads to low flood insurance coverage in Austria (e.g., Gruber, 2008; Hanger et al., 2017; Raschky, Schwarze, Schwindt, & Zahn, 2013). Thus, the disaster fund currently holds the most reliable data available for analysing household level natural hazard damages.

We consider claims associated with hydro‐meteorological hazards (flooding, landslides, debris flows) during the warm season (1 April to 30 November), and exclude damage from avalanches, snow pressure, and earthquakes. This covers 84% of all claims in the database. The data comprise the municipality (but not exact location), date, type of damage, and claimed costs. To estimate vulnerability and exposure at the macro‐scale of this analysis (c.f. Merz, Kreibich, Schwarze, & Thieken, 2010), we combine available data sets of indicators such as buildings, population, land use, and topography at the municipality level. Table 1 summarises the data used.

**Table 1:** Summary of the data used in this study

Description	Time period analysed	Resolution	Reference/source	Data used for
Point observations of precipitation (108 daily/72 sub‐daily gauges)	1990–2016	10 min/daily	Austrian national weather (ZAMG) and hydrographic (AHYD) services	Characterising precipitation in weather types
Daily discharge observations (61 gauges)	1990–2016	Daily	Austrian hydrographic service (AHYD), ehyd.gv.at	Calculating exceedance probabilities of discharge levels
Weather reviews and synoptic situation descriptions	1999–2017	Daily	Austrian national weather service (ZAMG)	Classifying weather types
gpard1 gridded precipitation dataset (quality controlled)	1990–2011	Daily, 1 x 1 km	Hofstätter et al. (2015)	Validating weather type classification, calculating average daily precipitation totals for all municipalities and estimating exceedance probabilities of precipitation events
INCA nowcasting system gridded precipitation (not quality controlled)	2004–2014	15 min, 1 x 1 km	Haiden et al. (2010)	
ERA‐interim reanalysis data (PSL, CAPE, geopotential, wind speed)	1979–2016	Daily	Dee et al. (2011)	Classifying weather types
Damage claims to the disaster relief fund	1990–2014 (available until 2016)	Daily	Departments of the provincial government of Styria (data.steiermark.at)	Analysing distributions among weather types and municipality clustering
Census data of the population in the Styrian municipalities	1991, 2001, 2011	10‐year		Municipality clustering
Digital elevation model	2014	10 x 10 m		Municipality clustering
CORINE land cover	2000	100 x 100 m	EEA (2017) (gis.epa.ie)	Municipality clustering
Shapefile of building outlines in the study area	2016	vector data	OpenStreetMap (openstreetmap.org)	Municipality clustering

*Note*. See Table S1 in Appendix S1, Supporting Information for a detailed list of gauges.

**Figure 1:**
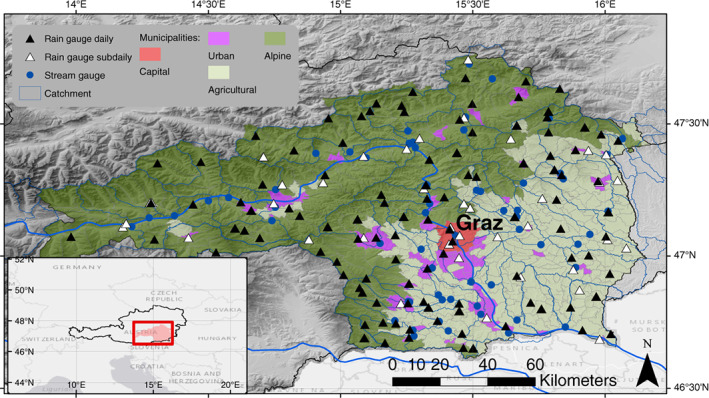
Study area, municipalities, gauge locations and catchments in the southeastern Alpine forelands of Austria. The shading of the municipalities denotes the group according to the cluster analysis

**Figure 2:**
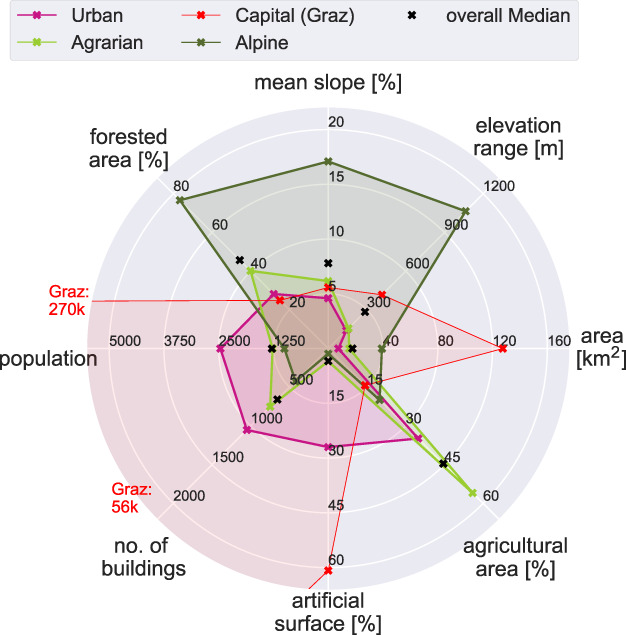
Factors of vulnerability and exposure in the four municipality clusters

The municipalities are divided into three groups of primarily agrarian, alpine, and urban character using principal component and hierarchical clustering analyses (Figure 1). The capital Graz builds a fourth cluster comprised of only one municipality with high population density. Figure 2 shows the properties of the clusters. Alpine municipalities are generally the steepest and most extensive, have considerable forestry, and cover the northwestern part of the study region. Agricultural municipalities lie in the southeastern part, with a similar population density as alpine municipalities, but considerably more buildings. The urban municipalities include county towns with higher population density, many buildings and artificial surfaces, and a third of agricultural area. Each of the four groups has a similar overall population of around 300,000 inhabitants.

**Figure 3:**
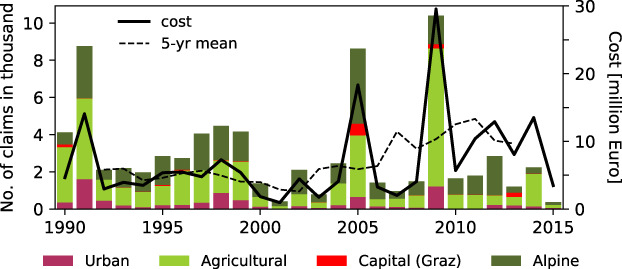
Number of damage claims from flooding, landslides, and debris flows per year and municipality cluster (bars, left axis) and total claim value per year (black solid line, right axis) as reported to the disaster fund 1990–2015 (1 April to 30 November)

Figure 3 shows the number and value of damage claims per year by municipality cluster. All costs were inflation adjusted to the Austrian consumer price index, with no further adjustments or normalizations. In total, a sum of 200 million € was claimed. A minimum claim threshold of 1,000€ superseded a model with deductibles and minimum payout sum in 2012 and so increased the official reporting threshold from approx. previous 600€. The high variability of hazard occurrence and decentralised handling of damage assessment make it difficult to quantify the effects of legislative changes. To avoid biases from the introduction of minimum reporting value, we remove all claims below 1,000€ from the sample before calculating trends. Simple trend analyses and Mann‐Kendall tests show no significant trends in annual number of claims or cost, but the average cost per claim has almost doubled from the first (1990–1999) to the most recent (2006–2015) decade of observations (factor 1.9). This value only changes slightly (factor 1.8) when the extreme years 1991 and 2009 are excluded. Calculating trends including claims below 1,000€ increases the factors to 2.3 and 2.5, respectively, indicating that the introduction of the minimum reporting value explains ~20% of the increased cost per claim in the full dataset.

Other reasons for an increased cost/claim ratio are potential increases in value of the objects damaged or the intensity of a hazard. Increased construction and property values in flood‐prone areas are often mentioned as a reason for rising flood damages, but this is not generally true as regional trends in development have to be considered (Fuchs, Keiler, & Zischg, 2015). For Styria, census data from 1991–2011 indicate the second lowest overall demographic change of all nine Austrian provinces, with decreasing population in 56% of municipalities (on average −2.6% per decade in the districts excluding Graz and Graz‐Surroundings) (Federal Government of Styria, 2014). Only the district surrounding Graz experienced a net increase in population (on average +10% per decade). The dynamics of land use change in Austria are among Europe's lowest, and the rate of artificial land take (0.21% p.a., corresponding to a total of 50 km^2^ over 2006–2012), is half that of the European average (EEA, 2017). Although changes are important on the local scale, the magnitude of these changes does not influence the clustering of municipalities.

For the distribution of incurred damages among convective and stratiform weather types, it can be assumed that vulnerable objects are equally exposed to the different weather types in any given year. We consider both the cost and number of claims. While claim occurrence indicates exposure, the accumulated costs on a particular day and/or place signal the severity of the event and level of vulnerability.

### 2.2 Precipitation and discharge

We use several data sources for a more robust analysis of precipitation observations. Sub‐daily precipitation data (10 min aggregated) and daily precipitation sums are available for 72 and 108 gauges, respectively. The gauges are operated by the Austrian weather service (ZAMG) and the hydrographic service of Styria (Figure 1). Daily (sub‐daily) records range from 1 to 27 (2 to 25) years with a median of 25 (13) years. See Tables S1–S3 in Appendix S1, Supporting Information for lengths and missing data in all station records. Rain gauge observations are used to assess sub‐daily precipitation characteristics of the weather types.

Furthermore we use the gridded, quality‐controlled dataset gpard1 (daily, 1990–2011) (Hofstätter et al., 2015), and the nowcasting product INCA, which blends station and radar observations (15 min aggregated to 1 day, 2004–2014) (Haiden et al., 2010). Both products are based on ZAMG gauge observations and are provided on a 1 km x 1 km grid. Gridded data are used to estimate daily precipitation totals over all municipalities on damage days.

No trends were identified in the sub‐daily precipitation data, however, studies found a decreasing trend in annual precipitation over the region (Masson & Frei, 2015). Agreement among data products is high on the daily scale, but correlations decrease towards sub‐hourly observations due to the high variability on small spatio‐temporal scales and storm movement. Furthermore, sub‐daily extremes and small‐scale phenomena are not well represented in gridded precipitation datasets (Hiebl & Frei, 2018; Schroeer, Kirchengast & O, 2017).

Average daily discharge data from gauges operated by the hydrographic service are available for 62 catchments ranging from 20 to 1,000 km^2^ (See Tables S1 and S4 in Appendix S1 for information on individual gauge records). Each municipality is associated with the relevant stream/river catchment. Administrative boundaries largely coincide with topography so that this mapping is unambiguous. The annual exceedance probability (AEP) of the daily discharge is calculated for each stream using Gumbel extreme value distributions fitted to the seasonal maxima over all available years. Kolmogorov‐Smirnov statistics support this choice for 100% of the gauges in summer, and for 99 and 92% of the gauges in spring and fall (autumn), respectively (95% confidence level). Information on anthropogenic modifications of the catchments was not available. For annual maximum daily discharge in Austrian discharge gauges, no change points were detected for the mean in the southeastern Alpine region, and significant change points in variance at two stations fall outside the study period (Villarini, Smith, Serinaldi, Ntelekos, & Schwarz, 2012). We address uncertainties in AEP by fitting the distribution to randomly selected two thirds of the data 1,000 times. The 90% confidence interval is then defined as the 5‐95th percentile range of AEPs calculated from each of these bootstrapped distributions.

The same method is applied to the seasonal maxima of daily rainfall calculated for each municipality using the gridded datasets to estimate AEPs of precipitation events (Kolmogorov–Smirnov test support a Gumbel distribution for all gauges except for one in fall). We follow Moftakhari et al. (2017) in classifying events with return intervals below 2 years as minor (AEP > 0.5), between 2 and 20 years (AEP ≤ 0.5 and AEP > 0.05) as major and ≥ 20 years (AEP ≤ 0.05) as extreme. These comparably low thresholds are justified by our interest in the cumulative effect of frequent events, the application to observed data only, and make calculations arguably more tractable, as statistical models are less sensitive to assumptions about the extreme tail behaviour (Serinaldi & Kilsby, 2014).

### 2.3 Weather types

The spatiotemporal coverage of sub‐daily precipitation observations is sparse compared to the spatiotemporal resolution of reported damages. This leads to high uncertainties when linking gauge‐based sub‐daily precipitation directly to damage in the municipalities. Through associating observed precipitation with larger‐scale synoptic conditions, we can utilise all available information and increase confidence in the character of precipitation events under different weather patterns. We first perform a circulation type classification based on daily ERA‐Interim data (1979–2016) (Dee et al., 2011) over the Greater Alpine Region (40.5–51.57°N, 3.0–20.25°E), using the COST Action 733 circulation type classification software (Philipp, Beck, Huth, & Jacobeit, 2016). The optimal classification scheme depends on the predictand (Huth, Beck, & Kucerova, 2016; Schiemann & Frei, 2010), here patterns of predominantly convective or stratiform precipitation. We apply principal component analysis and cluster analysis method (e.g., Prein et al., 2016) to sea level pressure, 700 hPa wind velocity, convective available potential energy (CAPE), and 500 hPa geopotential as indicators of atmospheric stability and seasonality, and to consider fast and slow moving systems. Codes for the synoptic situation over Austria were then collected from daily weather reports issued by the ZAMG since March 1, 1999.

Through merging the computationally identified weather classes with the weather types issued explicitly for Austria, we obtain a tailored classification of six weather types, WT1A, WT1B, WT1C, WT2, WT3, and WT4. WT1A to WT1C and WT4 are dominated by low gradient or high‐pressure situations, while WT2 and WT3 predominantly contain days of south and south‐westerly and north and north‐westerly flows, respectively. Positive CAPE anomalies and above‐average peak intensities distinguish the convective classes WT1A and WT1B occurring 1 June through 30 September. The difference is that WT1A sees low daily precipitation totals and wet spell durations mostly associated with single‐cell or multi‐cell storms, while extreme precipitation under WT1B is associated with disturbances and intense frontal precipitation and unstable atmospheric conditions, reaching higher precipitation totals and longer lasting events. WT1C is also dominated by low gradient situations, but CAPE and precipitation in general are lower in spring and fall. WT4 is dominated by persistent high‐pressure situations and negative precipitation anomalies. Rare precipitation events can occur in form of isolated thunderstorms on the warmest days in this class. Duration and daily precipitation totals are high in WT2, with only average peak intensities. In WT3, long wet spells, high daily precipitation totals, and low peak intensities prevail. Although dominated by large‐scale and stratiform precipitation, embedded convection at the onset of the precipitation event, particularly during the summer months, does occur.

**Figure 4:**
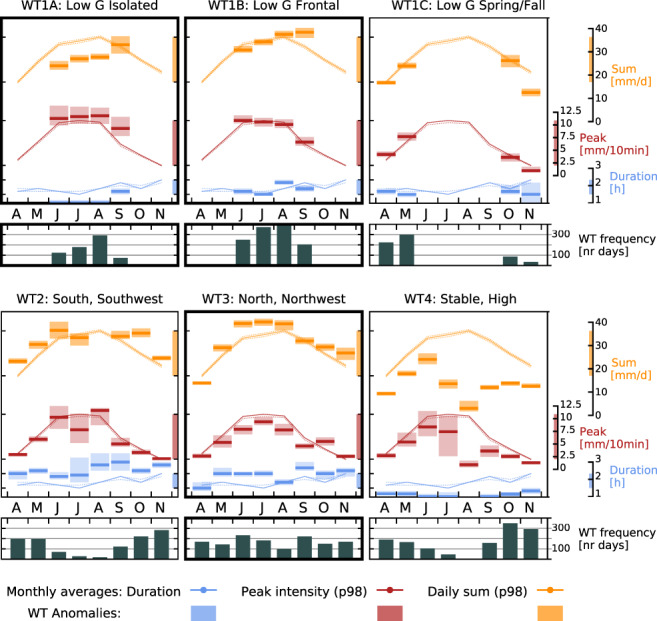
Weather type frequency (black bars, rows 2 and 4) and associated extreme precipitation (98th percentile, horizontal bars) for peak intensity (red), daily sum (yellow), and average wet spell duration (blue). Shadings show 90% confidence intervals. Full lines are identical in all panels and show the overall monthly climatological percentile values (full lines, dotted lines mark 90% confidence intervals). Note that the scales differ between colours

Figure 4 illustrates the frequency of each weather type during the study period and the weather‐type specific extreme (98th percentile) daily precipitation totals and 10‐min peak intensities and average wet spell duration (sum of 10 min intervals >0.1 mm) compared to the respective monthly percentile. These indicators were calculated from all rain gauges. WT‐specific discharges are provided in Figure S1 in Appendix S1.

## 3 RESULTS

### 3.1 Distribution of damage claims by weather types

**Figure 5:**
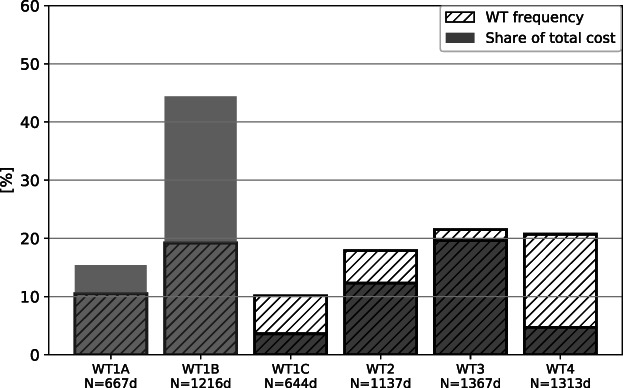
Relative frequency of days under all weather types (1990–2014, April to November) (hatched bars). Grey bars show the cumulated cost associated with each weather type relative of the total cost. Asterisks mark the relative frequency of damage reports within each weather type (number of days with reported damage divided by total number of weather type days)

Figure 5 shows the frequency (% days) of each weather type and the associated damage (% total reported cost). The predominantly convective weather types WT1A and WT1B show over‐proportional damage shares (15 and 43%, respectively), whereas little damage is reported during WT1C and WT4. ~12% of the total cost is attributed to WT2, while the share of damage under WT3 is approximately proportional to its occurrence (~20%).

**Figure 6:**
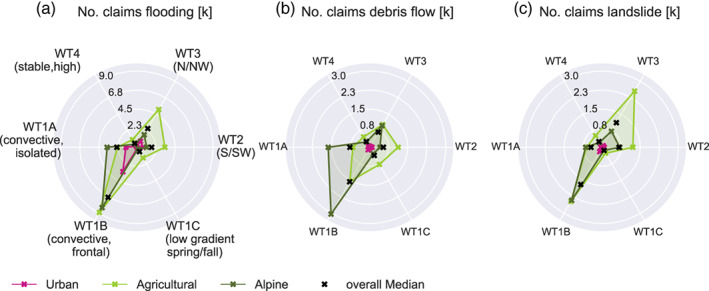
Number of claims to the disaster fund due to (a) flooding, (b) debris flows, and (c) landslides in rural, Alpine, and urban municipalities in the different weather type groups. Graz is not included due to the small numbers in claims. Note the different axis scaling in (a) and (b, c)

Generally, most claims are caused by flooding (~62%), followed by landslides (~20%) and debris flows (~18%). Figure 6 splits the claims by cause and municipality group. Graz is not included in this visualisation, because of the small total of 1,558 claims, of which ~90% are due to flooding. The breakdown shows an interesting division of the vulnerabilities of the different municipality groups to the weather types. WT1B is associated with most claims for all hazards and municipality groups except for landslides in the agricultural group. This is due to several large‐scale precipitation events in the region which caused exceptional amounts of landslides under WT2 and WT3 (Hornich & Adelwöhrer, 2010). Short but intense summer precipitation events under WT1A have less impact in this class, while alpine municipalities record the second largest frequency of both flood and debris flow claims in this weather type.

Precipitation processes interact with the basic topography and thus amplify vulnerabilities. While flooding can be expected anywhere in the study region, the agricultural group is prone to landslides due to its geological disposition, with over 5,000 km^2^, or half of the total area of the Province of Styria, classified in the highest of three landslide risk levels (Leopold, Draganits, Heiss, & Kovacs, 2013; Proske & Bauer, 2016). This part is also more directly influenced by southerly flow directions common under WT2, which occur in spring and fall when precipitation in the alpine municipalities in the higher northwestern parts can fall as snow and delay runoff. Furthermore, Alpine catchments are characterised by steep mountain topography and susceptibility to debris flows. Orography enhances convective precipitation in Alpine regions (Giorgi et al., 2016), a known trigger for debris flows.

Per capita claims in Graz and the urban municipalities are disproportionately low. Towns are usually not in steep areas prone to debris flows or landslides and most damage is caused by flooding under WT1B and WT1A. Because urban municipalities cover smaller areas, the probability of localised extreme precipitation hitting the area is smaller, yet if it does the consequences such as extreme runoff and surface water flooding occur immediately (c.f. Roodsari & Chandler, 2017; Sass et al., 2012; Syed et al., 2003), which is why urban areas are considered to be particularly vulnerable to flash floods (Guillén, Patalano, Garcia, & Bertoni, 2017; Kermanshah, Derrible, & Berkelhammer, 2017; Mahmood, Elagib, Horn, & Saad, 2017; Pereira, Diakakis, Deligiannakis, & Zezere, 2017). On the contrary, the area exposed is much larger in agricultural and alpine municipalities, and buildings are likely located closer to the hazard processes.

### 3.2 Annual exceedance probabilities of damage‐associated river discharge and precipitation

The previous section showed disproportionately high damage under convective weather types (WT1A, WT1B) compared to the incurred damage under stratiform weather types. To find out how this risk is reflected in hazard measures, we analyse the damage contributions under estimated minor, major, and extreme precipitation and discharge events and compare the patterns from convective weather types to the most damaging non‐convective weather type WT3.

**Figure 7:**
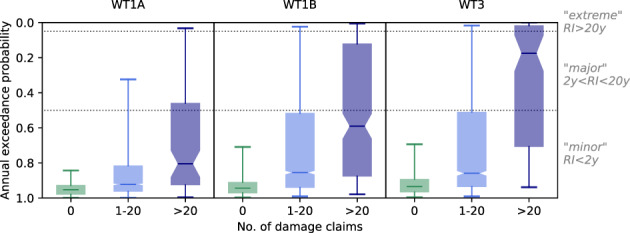
Annual exceedance probabilities of discharge levels on days with 0, 1–20, and > 20 reported claims under the convective WT1A and WT1B and the stratiform WT3. Whiskers denote the 5–95th percentile interval. Dotted horizontal lines mark the AEP thresholds applied in this study to define minor, major, and extreme events. Corresponding recurrence intervals (RI) are also given

The analysis of the annual exceedance probabilities (AEP) of river discharge shows considerable differences in the calculated flood risks among WT1A, WT1B, and WT3 (Figure 7). The AEP is shown separately for municipalities within catchments that were (a) not affected (no reported damage), (b) affected (1–20 damage claims), and (c) severely affected (>20 reported claims). This stratification indicates the severity of an event based on the impact and is independent of observations of precipitation or discharge.

Expectedly, AEP of discharge is always significantly lower on days with more damage claims than on days with moderate or no impact reported. However, under WT1A, AEPs only decrease slightly for moderate and high impact events. With medians in the minor flooding class, daily discharge observations rarely indicate the risk of damage under WT1A. Although discharge levels are significantly higher under WT1B, in ∼50% of the incidents the AEP of the closest stream gauge does not record unusual discharge even when more than 20 damage claims are reported in the catchment. On the contrary, high impact events under the stratiform WT3 are accompanied by major and even extreme discharge more regularly.

A possible reason for this is that widespread and long‐duration precipitation under WT3 allows more time for the rivers to react and the distance between damage location and stream gauge is less relevant under such conditions. In contrast, short‐duration, localised convective events might not have much effect on average daily discharges. Convective rainfall intense enough to cause damage might still not precipitate a large enough volume of water to raise water levels to a major or extreme level, especially in large rivers. If, additionally, the centre of a storm is located away from a stream gauge, this effect is even stronger, as runoff may be infiltrated or intercepted by the sewer system before reaching a stream.

Overall, minor discharge levels are observed on days accounting for 42.7–52.7% of the total reported cost. 15.9–18.6% and 5–17.7% of costs coincide with major and extreme river discharge levels, respectively. Less than 1% of the total cost associated with extreme discharge occurred under WT1A, in contrast to 2.3–6.8% under WT1B and 1.3–5.9% under WT3. For ∼25% of the total cost no discharge data is available for the respective catchment.

The differences in the distributions of hazard levels become more apparent when AEPs are considered relative to the weather type specific cost. Nuisance levels are associated with 69.2–73.6% of the damages under WT1A, with 41.1–52.5% under WT1B, and with only 27.3–34.8% under WT3. Likewise, 0.7–3.9% (WT1A), 5.3–15.4% (WT1B) and 6.7–22.9% (WT3) of weather type specific cost can be linked to extreme discharge.

**Figure 8:**
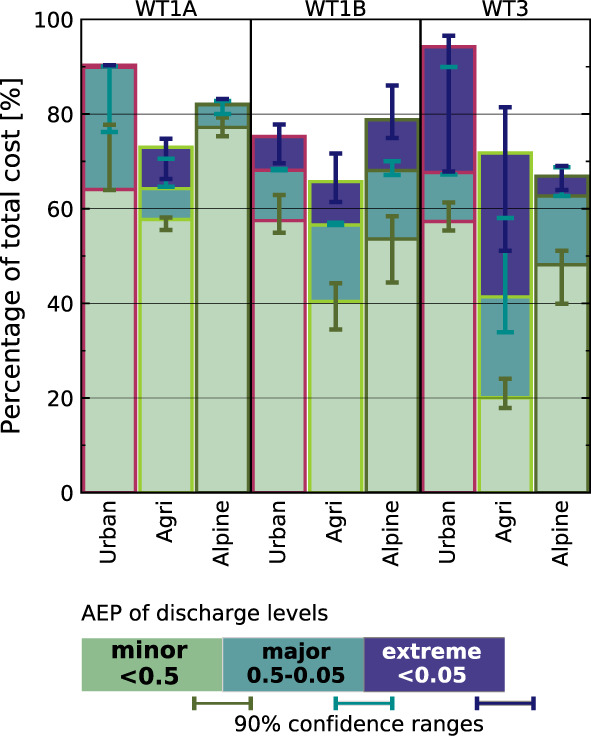
Relative proportion of claimed loss by annual exceedance probability of river discharge in each catchment. Green (light blue, dark blue) bars indicate minor (major, extreme) discharge levels. The proportions are shown individually for the total payout sums in urban, agricultural, and alpine municipalities under weather types WT1A, WT1B, and WT3. Error bars indicate the 90% confidence intervals of the individual contributions, that is, to be interpreted as the possible upper ends of each colour band. Space is left blank where no discharge data are available. Figure after Moftakhari et al. (2017)

Figure 8 shows the AEPs of discharge relative to the WT‐specific cost in the different municipality groups. Extreme discharge most frequently occurs in agricultural municipalities, particularly under WT3, where also the share of nuisance flooding is low (~20% of the cost). Also in urban municipalities, up to 20% of damage is associated with extreme discharge, albeit large uncertainty arises from the smaller sample. In alpine and urban municipalities under WT1A, daily discharge data almost never reach extreme levels. No significant differences are observed between municipality groups under WT1B.

The large share of cost associated with nuisance‐level events suggests that streamflow is not well correlated with large parts of the damage data. This can be either because damage was caused despite low discharge levels in form of nuisance flooding or surface water flooding, or because the discharge is not correctly observed. E.g., discharges used are those associated with the day of the reported claim and do not include delayed overland runoff. Although the data do not allow for disentangling of these effects, robust differences among weather types indicate that the risk of impacts under convective WT1A and, to lesser extent, frontal convective WT1B, is particularly underestimated in daily discharge data.

Precipitation is more directly linked to surface water flooding and AEPs of daily precipitation totals might better indicate the risk from convective extremes than available discharge data. Also here, minor events contribute 63.6–73% to the overall reported cost. Major and extreme level precipitation is observed in 18.5–19.7% and 6.5–14.8%, respectively. The proportion of total cost associated with extreme daily precipitation differs between convective (WT1A: 0.7–1.1%, WT1B: 2.5–6.2%) and stratiform (WT3: 9.9–11.1%) weather types. Within the weather type specific cost, 4.0–6.9% (WT1A), 5.6–14.1% (WT1B), and 16.7–29.0% (WT3) can be linked to extreme daily precipitation.

**Figure 9:**
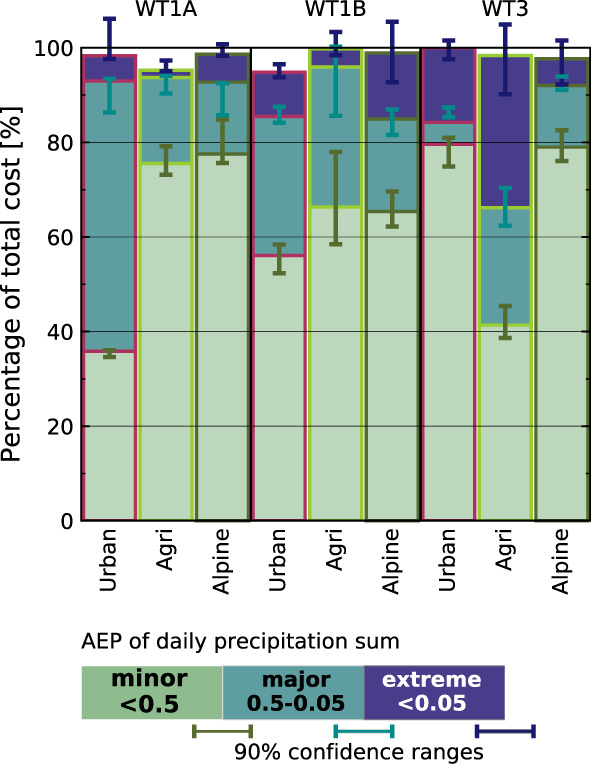
Relative proportion of claimed loss by annual exceedance probability of daily precipitation totals in the respective municipalities. Green (light blue, dark blue) bars indicate minor (major, extreme) precipitation totals. The proportions are shown individually for the total payout sums in urban, agricultural, and Alpine municipalities under weather types WT1A, WT1B, and WT3. Error bars indicate the 90% confidence intervals of the individual contributions, that is, to be interpreted as the possible upper ends of each colour band. Overshooting error bars can thus occur due to the stacked layout. Space is left blank where no precipitation data are available. Figure after Moftakhari et al. (2017)

Figure 9 shows the shares of the event magnitudes within each weather type and municipality group. Again, the agricultural municipalities see the lowest share of nuisance level events under WT3, whereas this fraction is particularly high under the isolated convective WT1A. In alpine municipalities, the group most vulnerable to WT1A, precipitation data better indicates the risk of damage than discharge data (5.9 vs. 0.1% of cost can be attributed to extreme levels of precipitation and discharge, respectively), although part of this might be due to better data coverage (2% of costs coincide with missing precipitation data).

In summary, both precipitation and discharge data agree quite well on the damage contributions under minor, major, and extreme events. For one half to two thirds of the total cost, observational data indicate only minor hazard levels, and this effect is particularly distinct in convective WTs even when major impacts were reported.

## 4 DISCUSSION

### 4.1 Discussion of uncertainties and limitations

The results showed that convective weather types are associated with frequent damage claims and that associated precipitation and discharge events often do not indicate this risk. Several sources of uncertainties come into play when interpreting these results.

The high number of damage reports that cannot be linked to significant precipitation or discharge levels indicates that extremes under WT1A and WT1B are indeed not well represented in observations. Under WT3, extremes from large‐scale precipitation patterns, which can be approximated with some confidence within the study region's observation network, seem to be better captured. Still, a high share of damage can only be linked to nuisance hazard levels. The share of nuisance level events is lowest in agricultural municipalities and particularly high in alpine municipalities. This indicates that the range of explanatory power of observations is smaller in the mountainous topography with secluded valleys in the alpine municipalities.

Increasing availability of precipitation data on small spatio‐temporal scales from high‐resolution networks and improving radar data will help to overcome these issues in the future. To understand damage fingerprints from convective and stratiform precipitation, better information is also needed on location and eligibilities of damages, and on procedural changes in claim handling. This would allow distinguishing damage from surface water flooding away from watercourses from flooding from overflowing streams. Furthermore, weather type specific exposures could be explicitly considered. For example, hail is associated with intense convective events (Nisi, Martius, Hering, Kunz, & Germann, 2016) and particularly with cold fronts during early summer (Schemm, Nisi, Martinov, Leuenberger, & Martius, 2016). Non‐availability of hail damage data likely leads to undervaluing the damage potential from convective patterns especially in agricultural municipalities. If exposure and vulnerability are generally similar to the analysed region‐wide household level data, the non‐availability of these data, for example, of damages to small businesses has little effect on the presented results of relative contributions.

Large uncertainty ranges in damage cost attributable to extreme discharge events highlight the difficulty in confidently assessing low frequency return levels. This is even though the threshold for extremes of annual exceedance probability of 0.05 is comparably low and the lengths of observational data series exceed the targeted return level. This underlines how more recent concepts such as the flood peak ratio, which avoids the calculation of large return intervals by setting observed flood peaks in relation to a 10‐year flood event (Czajkowski, Cunha, Michel‐Kerjan, & Smith, 2016), can help the assessment of extreme flood events.

Uncertainties also arise from changes in vulnerability and exposure over time. Regarding the presented results, the main question is whether implemented actions and measures have had divergent effects with respect to convective or stratiform hazards. Exposure and vulnerabilities might be weather type specific among sectors, or for direct and indirect damages. Such differences could not be considered here and need further research. After catastrophic events in 2005, the city of Graz initialized a 65 million €, 10‐year Special Programme—The Streams of Graz, investing in protection measures, retention areas, renaturalization, and educational advertising. Similarly, with expenditures of 27 million € per annum over 2000–2005, and 40 million € per annum 2005–2013, the province of Styria continuously invests in flood protection measures (Hornich, Zenz, Hammer, & Reischl, 2014). Construction projects often run for several years, and sometimes high‐risk land is purchased to incorporate it into the floodplain instead of building protection measures, thus the avoided impact difficult to assess. The fact that there is no obvious indication of damage reduction in the data analysed here calls for a deeper analysis of the effectiveness and temporal dimensions of such adaptation efforts. Effects of public awareness campaigns and private adaptation are generally hard to quantify (Aerts et al., 2018), but first studies show how disastrous events trigger adaptation (Kreibich et al., 2017). So far, these studies focus on large fluvial floods. Extending this research offers an opportunity to find out how various adaptation measures are effective in reducing impacts from large‐scale events and localised extremes, and stratiform or convective precipitation, respectively.

### 4.2 Implications for future risk assessment

Data on past losses include comprehensive information on damage from both frequent and rare events arising from convective and stratiform conditions. The large database analysed here helps to quantify the respective contributions and delivers insights into impacts related to convective precipitation events. This is important because many risk assessments, which often build on exceedance probabilities of inundation depths and coarse input data, might underestimate the risk from localised convective precipitation events as well as the cumulative effects from frequent nuisance level events.

This analysis showed that ∼60% of all damage occurred under synoptic conditions favouring convective precipitation with high sub‐daily intensities. For one half to two thirds of these incidents, neither the observed stream discharge nor the daily precipitation indicate this risk.

It is interesting that the contribution of extreme level events to overall damage under the predominantly stratiform WT3 (∼23%) is close to the estimates of how much extreme events contribute to total exposure (∼20%) in US coastal cities by Moftakhari et al. (2017). Although the scope and setting of the studies differ, this supports the hypothesis that large‐scale extremes are more easily captured in observational data and future flood risk assessments.

Hanger et al. (2017) find that “generally, governments focus more on large‐scale flood protection opportunities than on incentivizing private risk‐reduction behaviour”. Our results underline why focusing on large scale events is not enough to reduce impacts from extreme precipitation. Annual frequencies of weather types do not show significant changes over the study period, except for a slight increase in frequency of WT1B days (0.65 days per year, 90% confidence level). Extreme convective precipitation intensities are highly sensitive to temperature in southeastern Austria (Schroeer & Kirchengast, 2017) and are generally expected to increase with global warming (Zhang, Zwiers, Li, Wan, & Cannon, 2017). Although trends in sub‐daily extreme precipitation cannot be robustly detected at this point, this means that both the frequency of convective days and the intensity of precipitation on these days could increase in the future. It is thus important to facilitate private risk‐reduction behaviour to increase resilience to localised impacts and surface water flooding, against which technical measures along streams are less effective.

Most regular annual payouts of the disaster fund are spent on damage in rural and less densely populated areas. Claims per capita are much smaller in urban areas. Potential reasons for this are the smaller areas exposed and thus lower probability of an event occurring exactly here, and the fact that protection measures effectively protect a larger number of people than in dispersed settlements. Interesting differences between rural and urban areas regarding risk perception are observed also by Fuchs et al. (2017). They compare the perception and adaptation of inhabitants of a rural region susceptible to large‐scale river floods, and an urban area prone to flash floods. They find a lower individual disposition to pro‐actively invest in adaptation measures in the rural population exposed to large scale events, even though several floods occurred in the recent past. What is more, state and administrative authorities were blamed for unsatisfactory levels of protection measures. The willingness to accept human behaviour and development as risk factors and to individually adapt was higher in the urban area threatened by flash floods.

Even though the different socio‐cultural backgrounds must be considered, these findings relate to our results because they point towards the difficulties that may arise when developing adaptation strategies. It underlines the importance of differentiating the hazard processes as well as the socio‐economic context of the exposed area. Our results emphasise that private risk reduction behaviour is important to complement publicly funded structural measures concentrating on watercourses.

In the alpine municipalities, debris flows also cause a considerable proportion of the damage. In a modelling study, Meissl et al. (2017) reveal possible implications for steep mountain catchments in Alpine environments. Continued warming reduces the days with critical antecedent soil moisture conditions but increases mobilizable dried up litter. Hence, the consequences of intensified convective precipitation extremes could lead to rarer, but more intense debris flows.

Here, we established a relationship between critical circulation types and vulnerability factors using observed data. Such an approach, for example, through assessing larger‐scale trends in severe storm environments (Púčik et al., 2017), can support identifying future damage potential using climate models, where simulation of small‐scale extreme precipitation is improving, but considerable uncertainties remain (e.g., Olsson, Berg, & Kawamura, 2014). However, also changes of circulation types over the Alpine region need further research and uncertainties remain particularly in summer (Rohrer, Croci‐Maspoli, & Appenzeller, 2017).

## 5 CONCLUSION

We analysed a large database of damage claims in combination with several observational hydro‐meteorological datasets to identify contributions from different weather types and assess their reflection in hazard intensity measures. Observed and projected intensifications of convective precipitation and the associated high damage potential make this issue relevant to risk management activities. While at a specific location the event may be considered rare, impacts from convective storms occur more frequently at regional scales. The accumulated cost contributes significantly to the damage cost from natural hazards. Weather types indicate the character of the hazard event even when sub‐daily observations are not available at the damage location.

The present analysis showed that more than half of the damage from hydro‐meteorological hazards in the warm season (1 April to 30 November) can be attributed to synoptic situations favouring convective precipitation. In 50–60% of these events, associated river discharge levels are not considered to be extreme (AEP < 0.05). Under stratiform conditions, discharge reaches extreme levels more frequently, yet ∼40% of the damage is attributed to only minor discharge levels.

The generally high proportion of minor hazard levels indicates that convective precipitation, nuisance level events, and surface water flooding all significantly burden the disaster fund. Better resolved data on discharge, precipitation, and the location of damages, are needed to accurately ascribe damages to these processes. Large uncertainty ranges in AEP of extreme events underline the difficulty of accurately estimating return levels of extreme events from limited observations.

This initial study showed that identifying the risk‐prone environment through merging a “top‐down” weather typing approach with ground based observational data is beneficial for data‐limited analyses. It allows identifying the different natures of damaging precipitation events through their atmospheric drivers and local impacts and so improves our understanding of region‐ and context‐specific vulnerabilities.

## Supporting information


**Appendix S1** Supporting Information
**Figure S1.** Average monthly discharge anomalies associated with the different weather types over all stream gauges. Horizontal green lines show the average weather‐type specific deviation from the long‐term climatological mean (horizontal black lines); green boxes denote the 90% confidence intervals of the sample means.
**Figure S2.** Distribution of claims by municipality group and weather type, full sample
**Table S1.** Summary statistics of gauge records
**Table S2.** Information rain gauges (sub‐daily precipitation)
**Table S3.** Information rain gauges (daily precipitation). List includes 72 gauges from Table S 2, but record lengths are given for the daily observation records. Daily observations are available from an additional 80 rain gauges.
**Table S4.** Information stream gauges (daily observations)
