# Supplementary material for: Quantifying damage contributions from convective and stratiform weather types: How well do precipitation and discharge data indicate the risk?
Source: J Flood Risk Manag. 2018 Oct 2;12(4):e12491. doi: 10.1111/jfr3.12491 (PMC7003482; doi:10.1111/jfr3.12491)
Supplement: Supplementary file 1 — Appendix S1 Supporting Information Figure S1. Average monthly discharge anomalies associated with the different weather types over all stream gauges. Horizontal green lines show the average weather‐type specific deviation from the long‐term climatological mean (horizontal black lines); green boxes denote the 90% confidence intervals of the sample means. Figure S2. Distribution of claims by municipality group and weather type, full sample Table S1. Summary statistics of gauge records Table S2. Information rain gauges (sub‐daily precipitation) Table S3. Information rain gauges (daily precipitation). List includes 72 gauges from Table S 2, but record lengths are given for the daily observation records. Daily observations are available from an additional 80 rain gauges. Table S4. Information stream gauges (daily observations) [file JFR3-12-e12491-s001.pdf]

Supplementary information to Schroeer, K. and Tye, M.R.: Quantifying damage contributions from convective and stratiform weather types: how well do precipitation and discharge data indicate the risk?

Contents:

1. Figure S1: Discharge anomalies associated with weather types

2. Figure S2: Distribution of claims by municipality group and weather type, full sample

2. Table S1: Summary statistics of gauge records

3. Table S2: Information rain gauges (sub-daily precipitation)

4. Table S3: Information rain gauges (daily precipitation)

5. Table S4: Information stream gauges (daily observations)

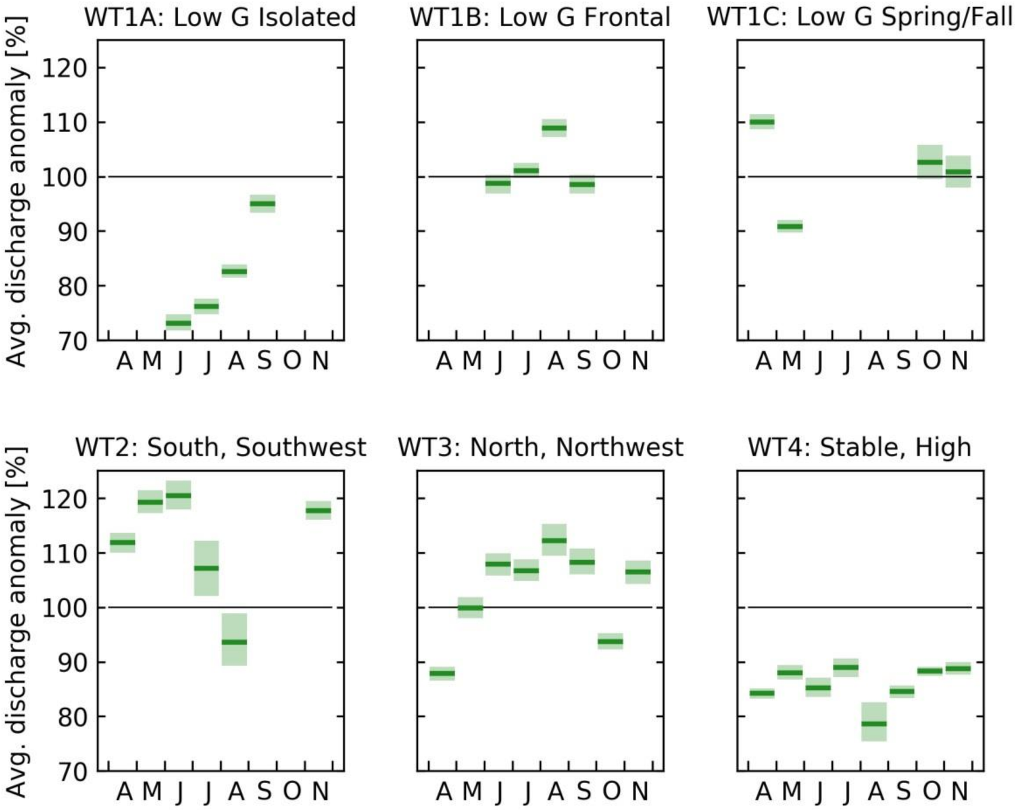

Figure 1: Average monthly discharge anomalies associated with the different weather types over all stream gauges. Horizontal green lines show the average weather-type specific deviation from the long-term climatological mean (horizontal black lines); green boxes denote the 90% confidence intervals of the sample means.

Table S 1: Summary statistics of gauge records

	**Number gauges**	**Median record length**	**Record lengths 5th and 95th percentile**	**Median Missing data study period**	**Missing data study period 5th and 95th percentile**
Sub-daily precipitation	72	13	[6.2, 22.3]	0.4	[0.0, 6.6]
Daily precipitation	108	40	[17, 114]	0.0	[0.0, 4.0]
Stream gauges	61	40	[20, 63]	0.0	[0.0, 0.0]

Table S 2: Information rain gauges (sub-daily precipitation)

	First observation	Last observation	Record length (years)	Missing data during study period (1990-2015) [%]	Provi-der	Lat	Lon
1	1992-08-29 00:10:00	2014-12-26 23:50:00	22.3	6.3	ZAMG	46.69	15.98
2	1992-08-29 00:10:00	2014-12-26 23:50:00	22.3	1.8	ZAMG	47.11	14.18
3	1992-08-29 00:10:00	2014-12-26 23:50:00	22.3	1.7	ZAMG	47.07	15.59
4	1992-08-29 00:10:00	2014-12-26 23:50:00	22.3	1.6	ZAMG	47.08	15.45
5	1993-02-16 13:30:00	2014-12-26 23:50:00	21.9	3.3	ZAMG	47.20	15.47
6	1993-07-29 11:10:00	2014-12-26 23:50:00	21.4	1.8	ZAMG	47.07	14.42
7	1994-10-24 13:30:00	2014-12-26 23:50:00	20.2	1.6	ZAMG	46.82	15.23
8	1994-10-24 13:10:00	2014-12-26 23:50:00	20.2	1.2	ZAMG	47.28	15.98
9	1997-02-17 12:40:00	2014-12-26 23:50:00	17.9	1	ZAMG	47.45	15.30
10	1997-09-01 00:10:00	2014-12-26 23:50:00	17.3	0.5	ZAMG	47.20	14.75
11	1999-08-16 09:30:00	2014-12-26 23:50:00	15.4	0.4	ZAMG	46.95	15.88
12	2001-11-08 16:10:00	2014-12-26 23:50:00	13.1	0.9	ZAMG	47.27	14.78
13	2002-07-31 14:20:00	2014-12-26 23:50:00	12.4	0.7	ZAMG	47.18	15.49
14	2003-04-08 16:10:00	2014-12-26 23:50:00	11.7	0.4	ZAMG	46.87	15.90
15	2003-11-03 12:10:00	2014-12-26 23:50:00	11.2	0.3	ZAMG	46.77	15.55
16	2004-10-28 14:50:00	2014-12-26 23:50:00	10.2	3.6	ZAMG	47.41	15.25
17	2004-10-31 20:10:00	2014-12-26 23:50:00	10.2	3.3	ZAMG	47.07	15.09
18	2004-11-09 12:10:00	2014-12-26 23:50:00	10.1	1	ZAMG	47.44	15.64
19	2007-05-09 10:00:00	2014-12-26 23:50:00	7.6	2	ZAMG	47.03	16.08
20	2007-05-08 08:40:00	2014-12-26 23:50:00	7.6	0.3	ZAMG	47.12	15.71
21	2007-07-20 17:00:00	2014-12-26 23:50:00	7.4	0.9	ZAMG	47.20	14.29
22	2007-07-24 12:00:00	2014-12-26 23:50:00	7.4	0.1	ZAMG	47.12	14.19
23	2007-11-07 10:00:00	2014-12-26 23:50:00	7.1	0.2	ZAMG	47.05	15.41
24	2008-09-05 14:50:00	2014-12-26 23:50:00	6.3	0.4	ZAMG	47.26	15.31
25	2008-08-25 12:30:00	2014-12-26 23:50:00	6.3	0.3	ZAMG	46.99	15.45
26	2008-11-10 15:40:00	2014-12-26 23:50:00	6.1	0.4	ZAMG	47.40	15.94
27	2012-10-04 13:20:00	2014-12-26 23:50:00	2.2	0.6	ZAMG	47.38	15.09
28	1982-06-01 07:00:00	2015-01-28 06:20:00	32.7	6.5	AHYD	47.33	15.81
29	1990-04-04 08:00:00	2015-01-01 00:00:00	24.8	24.4	AHYD	47.57	14.24
30	1999-01-01 07:00:00	2014-12-30 23:50:00	16	8.3	AHYD	46.65	15.50
31	1999-06-19 08:00:00	2015-01-27 23:50:00	15.6	0.1	AHYD	47.48	14.53
32	2000-01-01 07:00:00	2014-12-30 23:50:00	15	6.7	AHYD	47.14	15.06
33	2000-01-01 07:00:00	2015-01-01 06:50:00	15	1.7	AHYD	47.35	15.69
34	2000-01-01 07:00:00	2014-12-30 23:50:00	15	0.6	AHYD	46.81	15.33
35	2000-01-01 07:00:00	2014-12-30 23:50:00	15	0.1	AHYD	47.38	14.71
36	2000-01-01 07:00:00	2014-12-30 23:50:00	15	0	AHYD	47.06	14.88
37	2000-01-01 07:00:00	2015-01-01 06:50:00	15	0	AHYD	47.17	16.01
38	2000-01-01 07:00:00	2014-12-30 23:50:00	15	0	AHYD	46.90	15.26
39	2000-01-01 07:00:00	2015-01-01 06:50:00	15	0	AHYD	47.38	16.00
40	2000-07-01 07:00:00	2015-01-01 06:50:00	14.5	0.1	AHYD	47.49	15.92
41	2000-08-01 00:00:00	2014-12-30 23:50:00	14.4	0.1	AHYD	47.55	15.79
42	2000-08-01 00:00:00	2014-12-30 23:50:00	14.4	0	AHYD	47.15	15.24
43	2001-01-01 07:00:00	2014-12-30 23:50:00	14	0	AHYD	47.52	15.47
44	2001-01-01 07:00:00	2014-12-30 23:50:00	14	0	AHYD	46.79	15.45
45	2001-01-01 07:00:00	2014-12-30 23:50:00	14	0	AHYD	46.69	15.09
46	2001-01-01 07:00:00	2015-01-01 06:50:00	14	0	AHYD	47.22	15.76

47	2000-01-02 07:00:00	2013-06-20 13:10:00	13.5	0	AHYD	47.11	14.95
48	2001-10-01 07:00:00	2015-01-27 23:50:00	13.3	0	AHYD	47.74	15.48
49	2001-07-26 11:10:00	2014-10-16 06:50:00	13.2	3.2	AHYD	47.61	14.85
50	2002-01-01 07:00:00	2014-12-30 23:50:00	13	4.4	AHYD	47.20	14.28
51	2002-01-02 00:00:00	2014-12-30 23:50:00	13	0.4	AHYD	47.28	14.93
52	2002-01-01 07:00:00	2015-01-01 06:50:00	13	0	AHYD	46.85	15.97
53	2002-06-19 08:00:00	2015-01-18 10:40:00	12.6	0.6	AHYD	47.38	14.12
54	2002-06-19 08:00:00	2015-01-27 23:50:00	12.6	0.1	AHYD	47.51	14.83
55	2003-07-17 17:10:00	2014-12-30 12:50:00	11.5	0	AHYD	47.41	13.89
56	2003-10-01 00:00:00	2015-01-01 06:50:00	11.3	0	AHYD	46.92	15.68
57	2003-12-31 07:00:00	2014-12-30 23:50:00	11	0.2	AHYD	47.39	15.44
58	2003-12-31 07:00:00	2014-12-30 23:50:00	11	0	AHYD	47.17	14.66
59	2004-01-01 07:00:00	2015-01-01 06:50:00	11	0	AHYD	47.15	15.66
60	2004-01-01 07:00:00	2015-01-01 06:50:00	11	0	AHYD	47.29	16.05
61	2004-04-30 07:00:00	2014-12-30 23:50:00	10.7	0	AHYD	47.27	15.32
62	2004-10-01 07:00:00	2014-12-30 23:50:00	10.3	1.4	AHYD	47.11	14.60
63	2005-06-19 08:00:00	2015-01-27 23:50:00	9.6	0.1	AHYD	47.43	14.49
64	2005-07-01 07:00:00	2015-01-01 00:00:00	9.5	10.5	AHYD	47.66	14.98
65	2005-11-01 07:00:00	2014-12-30 23:50:00	9.2	0.7	AHYD	46.80	15.76
66	2005-12-31 07:00:00	2015-01-01 06:50:00	9	0.5	AHYD	47.06	16.01
67	2006-01-01 07:00:00	2015-01-01 00:00:00	9	0	AHYD	47.39	13.66
68	2006-06-19 08:00:00	2015-01-27 23:50:00	8.6	0	AHYD	47.59	14.63
69	2007-01-01 07:00:00	2014-12-30 23:50:00	8	0.4	AHYD	46.92	13.88
70	2007-09-17 13:50:00	2014-12-30 23:50:00	7.3	0	AHYD	47.11	15.42
71	2010-01-01 07:00:00	2015-01-01 06:50:00	5	0	AHYD	47.49	15.67
72	2010-11-16 15:40:00	2015-01-01 00:00:00	4.1	0	AHYD	47.37	13.72

Table S 3: Information rain gauges (daily precipitation). List includes 72 gauges from Table S 2, but record lengths are given for the daily observation records. Daily observations are available from an additional 80 rain gauges.

	First observation	Last observation	Record length (years)	Missing data during study period (1990-2015) [%]	Provi-der	Lat	Lon
1	1983-05-01 00:00:00	2017-02-07 00:00:00	33.8	0.3	ZAMG	47.55	15.24
2	1999-04-01 00:00:00	2017-02-07 00:00:00	17.9	0.8	ZAMG	47.63	15.83
3	2000-09-30 00:00:00	2017-02-06 00:00:00	16.4	0.3	ZAMG	47.52	14.95
4	2008-09-08 00:00:00	2017-02-07 00:00:00	8.4	0	ZAMG	47.60	15.67
5	1900-01-00 00:00:00	2014-12-31 00:00:00	115.1	0	AHYD	46.90	15.26
6	1901-01-01 00:00:00	2014-12-31 00:00:00	114.1	14.3	AHYD	46.93	15.67
7	1901-01-01 00:00:00	2014-12-31 00:00:00	114.1	5.3	AHYD	47.27	15.32
8	1901-01-01 00:00:00	2014-12-31 00:00:00	114.1	4	AHYD	47.22	15.39
9	1901-01-01 00:00:00	2014-12-31 00:00:00	114.1	0	AHYD	46.79	15.45
10	1901-01-01 00:00:00	2014-12-31 00:00:00	114.1	0	AHYD	47.17	16.01
11	1901-01-01 00:00:00	2014-12-31 00:00:00	114.1	0	AHYD	47.46	14.68
12	1901-01-01 00:00:00	2014-12-31 00:00:00	114.1	0	AHYD	46.82	15.45
13	1901-01-01 00:00:00	2014-12-31 00:00:00	114.1	0	AHYD	47.10	15.83
14	1901-01-01 00:00:00	2014-12-31 00:00:00	114.1	0	AHYD	47.07	14.69
15	1901-01-01 00:00:00	2014-12-31 00:00:00	114.1	0	AHYD	46.69	15.26
16	1901-01-01 00:00:00	2014-12-31 00:00:00	114.1	0	AHYD	47.06	14.30
17	1901-01-01 00:00:00	2014-12-31 00:00:00	114.1	0	AHYD	47.02	14.92
18	1901-01-01 00:00:00	2014-12-31 00:00:00	114.1	0	AHYD	46.99	15.21
19	1901-01-01 00:00:00	2014-12-31 00:00:00	114.1	0	AHYD	47.07	15.07
20	1901-01-01 00:00:00	2014-12-31 00:00:00	114.1	0	AHYD	46.81	15.87
21	1901-01-01 00:00:00	2014-12-31 00:00:00	114.1	0	AHYD	47.53	15.78
22	1901-01-01 00:00:00	2014-12-31 00:00:00	114.1	0	AHYD	47.36	14.47
23	1901-01-01 00:00:00	2014-12-31 00:00:00	114.1	0	AHYD	47.20	14.44
24	1901-01-01 00:00:00	2014-12-31 00:00:00	114.1	0	AHYD	47.67	15.47
25	1901-01-01 00:00:00	2014-12-31 00:00:00	114.1	0	AHYD	47.47	15.50
26	1901-01-01 00:00:00	2014-12-31 00:00:00	114.1	0	AHYD	47.53	15.08
27	1901-01-01 00:00:00	2014-12-31 00:00:00	114.1	0	AHYD	47.58	15.50
28	1901-01-01 00:00:00	2014-12-31 00:00:00	114.1	0	AHYD	47.28	15.97
29	1901-01-01 00:00:00	2014-12-31 00:00:00	114.1	0	AHYD	47.47	15.79
30	1901-01-01 00:00:00	2014-12-31 00:00:00	114.1	0	AHYD	47.21	15.82
31	1901-01-01 00:00:00	2014-12-31 00:00:00	114.1	0	AHYD	47.40	15.89
32	1902-01-01 00:00:00	2014-12-31 00:00:00	113.1	0	AHYD	47.40	14.82
33	1901-01-01 00:00:00	2006-04-30 00:00:00	105.4	0	AHYD	47.31	14.94
34	1913-01-01 00:00:00	2014-12-31 00:00:00	102.1	0	AHYD	47.27	14.71
35	1927-07-01 00:00:00	2014-12-31 00:00:00	87.6	0	AHYD	47.43	15.01
36	1929-06-01 00:00:00	2014-12-31 00:00:00	85.6	0	AHYD	46.80	15.76
37	1930-01-01 00:00:00	2014-12-31 00:00:00	85.1	0	AHYD	46.98	14.99
38	1930-01-01 00:00:00	2014-12-31 00:00:00	85.1	0	AHYD	46.93	15.01
39	1933-01-01 00:00:00	2014-12-31 00:00:00	82.1	0	AHYD	47.15	15.24
40	1936-01-01 00:00:00	2014-12-31 00:00:00	79.1	0	AHYD	46.65	15.46
41	1946-01-01 00:00:00	2014-12-31 00:00:00	69	0	AHYD	47.10	15.41

42	1947-03-01 00:00:00	2014-12-31 00:00:00	67.9	0	AHYD	47.17	14.66
43	1947-07-01 00:00:00	2014-12-31 00:00:00	67.5	0	AHYD	47.39	15.44
44	1948-01-01 00:00:00	2014-12-31 00:00:00	67	1.7	AHYD	47.01	14.53
45	1951-11-01 00:00:00	2014-12-31 00:00:00	63.2	0	AHYD	46.95	15.33
46	1952-01-01 00:00:00	2014-12-31 00:00:00	63	0	AHYD	47.66	15.70
47	1954-08-01 00:00:00	2014-12-31 00:00:00	60.5	4	AHYD	46.75	15.16
48	1955-01-01 00:00:00	2014-12-31 00:00:00	60	0	AHYD	46.71	16.02
49	1955-11-01 00:00:00	2014-12-31 00:00:00	59.2	0	AHYD	47.00	15.94
50	1971-01-01 00:00:00	2014-12-31 00:00:00	44	4	AHYD	47.11	14.60
51	1971-01-01 00:00:00	2014-12-31 00:00:00	44	0	AHYD	47.14	15.33
52	1971-01-01 00:00:00	2014-12-31 00:00:00	44	0	AHYD	47.44	16.05
53	1971-01-01 00:00:00	2014-12-31 00:00:00	44	0	AHYD	46.72	15.91
54	1961-01-01 00:00:00	2003-01-31 00:00:00	42.1	0	AHYD	47.20	14.28
55	1974-01-01 00:00:00	2014-12-31 00:00:00	41	0	AHYD	47.07	15.13
56	1974-07-01 00:00:00	2014-12-31 00:00:00	40.5	0	AHYD	47.07	13.98
57	1961-01-01 00:00:00	2001-01-31 00:00:00	40.1	9	AHYD	47.58	15.14
58	1975-01-01 00:00:00	2014-12-31 00:00:00	40	0	AHYD	46.74	15.62
59	1975-01-01 00:00:00	2014-12-31 00:00:00	40	0	AHYD	47.31	15.84
60	1975-01-01 00:00:00	2014-12-31 00:00:00	40	0	AHYD	47.38	15.79
61	1975-01-01 00:00:00	2014-12-31 00:00:00	40	0	AHYD	46.93	16.02
62	1977-01-01 00:00:00	2014-12-31 00:00:00	38	0	AHYD	47.07	16.00
63	1977-05-01 00:00:00	2014-12-31 00:00:00	37.7	0	AHYD	46.72	15.26
64	1971-01-01 00:00:00	2008-06-30 00:00:00	37.5	0	AHYD	47.35	14.31
65	1979-01-01 00:00:00	2014-12-31 00:00:00	36	0	AHYD	47.23	15.15
66	1980-01-01 00:00:00	2014-12-31 00:00:00	35	0	AHYD	47.08	15.21
67	1981-01-01 00:00:00	2014-12-31 00:00:00	34	0	AHYD	47.30	15.51
68	1981-01-01 00:00:00	2014-12-31 00:00:00	34	0	AHYD	46.82	15.72
69	1957-07-01 00:00:00	1990-12-31 00:00:00	33.5	0	AHYD	47.57	15.23
70	1982-01-01 00:00:00	2014-12-31 00:00:00	33	0	AHYD	47.46	15.99
71	1984-01-01 00:00:00	2014-12-31 00:00:00	32.7	0	AHYD	47.33	15.81
72	1971-01-01 00:00:00	2002-12-31 00:00:00	32	1.9	AHYD	46.85	15.97
73	1984-01-01 00:00:00	2014-12-31 00:00:00	31	0	AHYD	46.69	15.09
74	1984-01-01 00:00:00	2014-12-31 00:00:00	31	0	AHYD	47.20	15.17
75	1984-01-01 00:00:00	2014-12-31 00:00:00	31	0	AHYD	46.75	15.21
76	1984-01-01 00:00:00	2014-12-31 00:00:00	31	0	AHYD	46.67	15.17
77	1985-01-01 00:00:00	2014-12-31 00:00:00	30	12	AHYD	46.82	15.14
78	1975-01-01 00:00:00	2004-12-31 00:00:00	30	0	AHYD	46.76	15.37
79	1985-01-01 00:00:00	2014-12-31 00:00:00	30	0	AHYD	46.86	15.08
80	1986-10-01 00:00:00	2014-12-31 00:00:00	28.3	0	AHYD	46.83	15.31
81	1987-01-01 00:00:00	2014-12-31 00:00:00	28	0	AHYD	46.71	15.46
82	1987-01-01 00:00:00	2014-12-31 00:00:00	28	0	AHYD	46.92	15.10
83	1988-01-01 00:00:00	2014-12-31 00:00:00	27	0	AHYD	47.31	15.48
84	1988-01-01 00:00:00	2014-12-31 00:00:00	27	0	AHYD	46.74	15.09
85	1988-01-01 00:00:00	2014-12-31 00:00:00	27	0	AHYD	47.18	14.81
86	1990-01-01 00:00:00	2014-12-31 00:00:00	25	0	AHYD	47.49	15.67
87	1990-01-01 00:00:00	2014-12-31 00:00:00	25	0	AHYD	46.99	15.87
88	1991-01-01 00:00:00	2014-12-31 00:00:00	24	0	AHYD	47.11	14.95
89	1991-01-01 00:00:00	2014-12-31 00:00:00	24	0	AHYD	47.01	15.39
90	1991-01-01 00:00:00	2014-12-31 00:00:00	24	0	AHYD	47.37	15.34
91	1991-01-01 00:00:00	2014-12-31 00:00:00	24	0	AHYD	47.55	15.11
92	1994-01-01 00:00:00	2014-12-31 00:00:00	21	0	AHYD	46.65	15.50
93	1994-01-01 00:00:00	2014-12-31 00:00:00	21	0	AHYD	47.14	15.06
94	1994-01-01 00:00:00	2014-12-31 00:00:00	21	0	AHYD	47.22	15.33
95	1994-01-01 00:00:00	2014-12-31 00:00:00	21	0	AHYD	46.88	15.07
96	1994-01-01 00:00:00	2014-12-31 00:00:00	21	0	AHYD	46.82	15.25
97	1994-01-01 00:00:00	2014-12-31 00:00:00	21	0	AHYD	47.10	14.64
98	1994-01-01 00:00:00	2014-12-31 00:00:00	21	0	AHYD	47.15	14.85
99	1996-01-01 00:00:00	2014-12-31 00:00:00	19	0	AHYD	47.15	15.66
100	1997-01-01 00:00:00	2014-12-31 00:00:00	18	0	AHYD	47.35	15.69
101	1998-01-01 00:00:00	2014-12-31 00:00:00	17	0	AHYD	46.81	15.33

102	1998-01-01 00:00:00	2014-12-31 00:00:00	17	0	AHYD	46.92	13.88
103	1998-01-01 00:00:00	2014-12-31 00:00:00	17	0	AHYD	47.06	15.42
104	1998-01-01 00:00:00	2014-12-31 00:00:00	17	0	AHYD	47.20	14.62
105	1998-01-01 00:00:00	2014-12-31 00:00:00	17	0	AHYD	47.62	15.27
106	1999-03-01 00:00:00	2014-12-31 00:00:00	15.8	14.2	AHYD	47.49	15.92
107	1994-01-01 00:00:00	2002-02-28 00:00:00	14.4	0	AHYD	47.55	15.79
108	2011-12-01 00:00:00	2014-12-31 00:00:00	14	0	AHYD	47.52	15.45

Table S 4: Information stream gauges (daily observations)

	**First observation**	**Last observation**	**Record length (years)**	**Missing data during study period (1990-2015) [%]**	**Provi-der**	**Lat**	**Lon**
1	1951-01-01 00:00:00	2014-12-31 00:00:00	64	0	AHYD	47.21	14.54
2	1951-01-01 00:00:00	2013-12-30 00:00:00	63	0	AHYD	47.02	16.14
3	1951-01-01 00:00:00	2013-12-30 00:00:00	63	0	AHYD	46.96	15.89
4	1951-01-01 00:00:00	2013-12-30 00:00:00	63	0	AHYD	47.22	14.58
5	1951-01-01 00:00:00	2013-12-30 00:00:00	63	0	AHYD	47.38	15.09
6	1951-01-01 00:00:00	2013-12-30 00:00:00	63	0	AHYD	47.47	15.25
7	1951-01-01 00:00:00	2013-12-30 00:00:00	63	0	AHYD	46.96	15.35
8	1951-01-01 00:00:00	2013-12-30 00:00:00	63	0	AHYD	46.76	15.21
9	1951-01-01 00:00:00	2013-12-30 00:00:00	63	0	AHYD	46.72	15.27
10	1951-01-01 00:00:00	2013-12-30 00:00:00	63	0	AHYD	46.78	15.53
11	1961-01-01 00:00:00	2013-12-30 00:00:00	53	0	AHYD	47.21	16.09
12	1961-01-01 00:00:00	2013-12-30 00:00:00	53	0	AHYD	47.17	16.01
13	1961-01-01 00:00:00	2013-12-30 00:00:00	53	0	AHYD	47.28	15.69
14	1961-01-01 00:00:00	2013-12-30 00:00:00	53	0	AHYD	47.08	15.94
15	1961-01-01 00:00:00	2013-12-30 00:00:00	53	0	AHYD	47.11	14.21
16	1961-01-01 00:00:00	2013-12-30 00:00:00	53	0	AHYD	47.43	15.26
17	1961-01-01 00:00:00	2013-12-30 00:00:00	53	0	AHYD	46.81	15.52
18	1966-01-01 00:00:00	2014-12-31 00:00:00	49	0	AHYD	47.03	15.45
19	1966-01-01 00:00:00	2013-12-30 00:00:00	48	0	AHYD	47.18	15.67
20	1966-01-01 00:00:00	2013-12-30 00:00:00	48	0	AHYD	47.38	16.00
21	1966-01-01 00:00:00	2013-12-30 00:00:00	48	0	AHYD	47.19	14.75
22	1966-01-01 00:00:00	2013-12-30 00:00:00	48	0	AHYD	47.39	14.91
23	1966-01-01 00:00:00	2013-12-30 00:00:00	48	0	AHYD	47.39	15.03
24	1966-01-01 00:00:00	2013-12-30 00:00:00	48	0	AHYD	47.53	15.47
25	1966-01-01 00:00:00	2013-12-30 00:00:00	48	0	AHYD	47.05	15.15
26	1967-01-01 00:00:00	2013-12-30 00:00:00	47	0	AHYD	47.41	15.28
27	1968-01-01 00:00:00	2013-12-30 00:00:00	46	0	AHYD	47.04	15.76
28	1969-01-01 00:00:00	2013-12-30 00:00:00	45	0	AHYD	46.76	15.67
29	1971-01-01 00:00:00	2013-12-30 00:00:00	43	0	AHYD	47.43	15.27
30	1972-01-01 00:00:00	2013-12-30 00:00:00	42	0	AHYD	47.25	15.51
31	1974-01-01 00:00:00	2013-12-30 00:00:00	40	0	AHYD	46.71	15.79
32	1976-01-01 00:00:00	2013-12-30 00:00:00	38	0	AHYD	46.73	15.85
33	1977-01-01 00:00:00	2013-12-30 00:00:00	37	0	AHYD	47.29	16.09
34	1979-01-01 00:00:00	2013-12-30 00:00:00	35	0	AHYD	46.99	16.21
35	1980-01-01 00:00:00	2013-12-30 00:00:00	34	0	AHYD	47.17	15.62

36	1981-01-01 00:00:00	2013-12-30 00:00:00	33	0	AHYD	47.31	15.83
37	1981-01-01 00:00:00	2013-12-30 00:00:00	33	0	AHYD	47.29	15.84
38	1981-01-01 00:00:00	2013-12-30 00:00:00	33	0	AHYD	47.20	15.34
39	1982-01-01 00:00:00	2013-12-30 00:00:00	32	0	AHYD	46.72	15.41
40	1982-01-01 00:00:00	2013-12-30 00:00:00	32	0	AHYD	46.83	15.26
41	1982-01-01 00:00:00	2013-12-30 00:00:00	32	0	AHYD	47.33	16.05
42	1982-01-01 00:00:00	2013-12-30 00:00:00	32	0	AHYD	47.25	15.52
43	1984-01-01 00:00:00	2013-12-30 00:00:00	30	0	AHYD	47.15	15.67
44	1987-01-01 00:00:00	2013-12-30 00:00:00	27	4.7	AHYD	47.52	15.79
45	1987-01-01 00:00:00	2013-12-30 00:00:00	27	0	AHYD	47.37	16.12
46	1987-01-01 00:00:00	2013-12-30 00:00:00	27	0	AHYD	47.13	14.74
47	1987-01-01 00:00:00	2013-12-30 00:00:00	27	0	AHYD	47.16	15.32
48	1989-01-01 00:00:00	2013-12-30 00:00:00	25	0	AHYD	46.84	15.38
49	1989-01-01 00:00:00	2013-12-30 00:00:00	25	0	AHYD	46.83	15.26
50	1990-01-01 00:00:00	2013-12-30 00:00:00	24	0	AHYD	46.85	15.37
51	1990-01-01 00:00:00	2013-12-30 00:00:00	24	0	AHYD	46.90	15.49
52	1991-01-01 00:00:00	2013-12-30 00:00:00	23	0	AHYD	46.93	16.16
53	1991-01-01 00:00:00	2013-12-30 00:00:00	23	0	AHYD	47.48	15.47
54	1991-01-01 00:00:00	2013-12-30 00:00:00	23	0	AHYD	47.10	15.68
55	1993-01-01 00:00:00	2013-12-30 00:00:00	21	0	AHYD	46.89	15.57
56	1993-01-01 00:00:00	2013-12-30 00:00:00	21	0	AHYD	46.75	15.37
57	1994-01-01 00:00:00	2013-12-30 00:00:00	20	0	AHYD	47.25	14.76
58	1994-01-01 00:00:00	2013-12-30 00:00:00	20	0	AHYD	46.70	15.27
59	1995-01-01 00:00:00	2013-12-30 00:00:00	19	0	AHYD	47.03	15.30
60	2000-06-20 00:00:00	2013-12-30 00:00:00	13.5	0	AHYD	47.14	14.28
61	2003-09-02 00:00:00	2013-12-30 00:00:00	10.3	0	AHYD	47.15	14.37
